# Lower Rates of Hepatocellular Carcinoma Observed Among Cannabis Users: A Population-Based Study

**DOI:** 10.7759/cureus.24576

**Published:** 2022-04-28

**Authors:** Ahmed ElTelbany, George Khoudari, Yasser Al-Khadra, Arthur McCullough, Naim Alkhouri

**Affiliations:** 1 Internal Medicine, Cleveland Clinic, Cleveland, USA; 2 Gastroenterology and Hepatology, MedStar Georgetown University Hospital, Washington DC, USA; 3 Cardiology, Southern Illinois University School of Medicine, Springfield, USA; 4 Gastroenterology and Hepatology, Cleveland Clinic, Cleveland, USA; 5 Fatty Liver Program, Arizona Liver Health, Pheonix, USA

**Keywords:** hepatocellular carcinoma, cannabis (marijuana), cannabis research, liver cancer directed therapies, cannabidiol oil, population based research, cannabis use

## Abstract

Background: Hepatocellular carcinoma (HCC) is one of the most common malignancies worldwide and the fourth leading cause of cancer deaths in the world. The association between HCC and cannabis has been identified in mice; however, to our knowledge has not been identified in humans. Therefore, we aim to investigate the relation between HCC and cannabis use in humans.

Methods: Using data from the National Inpatient Sample (NIS) database between 2002 and 2014, we identified the patients with HCC and cannabis use diagnosis using the International Classification of Disease 9^th^ version codes (ICD-9). Then, we identified patients without cannabis use as the control group. We adjusted for multiple potential confounders and performed multivariable logistic regression analysis to determine the association between cannabis abuse and HCC.

Results: A total of 101,231,036 patients were included in the study. Out of the total, 996,290 patients (1%) had the diagnosis of cannabis abuse versus 100,234,746 patients (99%) in the control group without cannabis abuse. We noticed that patients with cannabis abuse were younger (34 vs 48 years), had more males (61.7% vs 41.4%) and more African Americans (29.9% vs 14.2%) compared with the control group (P<0.001 for all). Besides, patients with cannabis use had more hepatitis B, hepatitis C, liver cirrhosis, and smoking, but had less obesity and gallstones, (P<0.001 for all). Using multivariable logistic regression, and after adjusting for potential confounders, patients with cannabis abuse were 55% less likely to have HCC (adjusted Odds Ratio {aOR}, 0.45, 95% Confidence Interval {CI}, 0.42-0.49, P<0.001) compared with patients without cannabis abuse.

Conclusion: Based on our large database analysis, we found that cannabis use patients were 55% less likely to have HCC compared to patients without cannabis use. Further prospective studies are needed to assess the role of cannabis use on HCC.

## Introduction

Cannabis is among the most abused psychoactive substances in the United States. It is considered only second to alcohol and tobacco in terms of global consumption [1]. It is primarily used for recreational purposes, but recently it has been considered for clinical applications. According to the World Health Organization (WHO), it is currently estimated that between 128-232 million people worldwide, and over 22 million individuals in the United States of America (USA), aged 12 years and above use cannabis with approximately 3.6 million Americans considered daily or near-daily users [1,2]. Recent legislation across different states in the USA has legalized the recreational use of marijuana, which is expected to grow the number of marijuana users even further. Despite its widespread use, the clinical benefits of marijuana remain to be further investigated.

Hepatocellular carcinoma (HCC) accounts for the majority of primary liver cancers. Globally it is the fourth leading cause of cancer-related death and ranks sixth in terms of incidence [3]. According to the WHO, the incidence of HCC is expected to increase until 2030, with overestimates in excess of 1 million patients death from liver cancer [4]. In the US, the rate of death from liver cancer increased by 43% (from 7.2 to 10.3 deaths per 100,000) between 2000 and 2016 [5]. After pancreatic cancer, liver cancer is the most lethal tumor with an estimated 5-year survival of 18% [6]. Hepatitis B or C virus infection or alcohol abuse accounts for the majority of HCC patients. With the advent of universal hepatitis B virus (HBV) vaccination and direct antiviral agents against hepatitis C virus (HCV), it is expected that nonalcoholic fatty liver disease (NAFLD) will soon be the leading cause of HCC [7]. Current treatment options are only applicable to the early stages of tumor development and include tumor resection, liver transplantation, chemo-embolization, and sorafenib administration. Unfortunately, half of the patients will experience tumor recurrence [8].

In recent years, several clinical trials have tested the efficacy of novel agents that selectively target the carcinogenic process in HCC. Unfortunately, no relevant improvement in the prognosis of patients with HCC has been achieved so far [9]. In recent years, the medical and health-related applications of cannabidiol (CBD), a chemical found in cannabis have garnered tremendous attention [10]. To the best of our knowledge, no association has been identified between HCC and cannabis use in humans. Thus, we sought to use the largest publicly available in-patient database to analyze the relation between cannabis use and its effect on the HCC population. This article was previously presented as a meeting abstract at the 2019 DDW Annual Scientific Meeting on May 18th, 2019.

## Materials and methods

Data source

The national inpatient sampling (NIS) is a publicly available identified database of hospital discharges in the US, containing data from approximately 8 million hospital stays that were selected using a complex probability sampling design and the weighting scheme recommended by the Agency for Healthcare Research and Quality which is intended to represent all discharges from nonfederal hospitals. Each record includes one primary diagnosis and up to 15 secondary diagnoses.

Study population

For this study, we identified all patients from 2002 to 2014 who had the diagnosis as cannabis users using the International Classification of Disease, Ninth Edition, Clinical Modification (ICD-9-CM) code (304.3, 304.30, 304.31, 304.32, 304.33, 305.2, 305.20, 305.21, 305.22, 305.23). Also, we identified patients with the diagnosis of HCC (ICD-9-CM) code (155.0). Using the Clinical Classification Software codes provided by the Healthcare Cost and Utilization Project and the Elixhauser Comorbidity Index, co-morbidities were appointed via ICD-9 codes.

Outcomes

After Identifying patients with cannabis use, we investigated the risk factors associated with HCC. The primary outcome was the association between cannabis users and HCC. The secondary outcomes were the risk factors associated with HCC. Supplementary Table 4 identifies co-morbidities from the Elixhauser comorbidity index used in the analysis. Supplementary Table 5 identifies ICD-9 codes used for other co-morbidities.

Statistical analysis

Continuous variables were expressed as weighted mean ± standard deviation, and frequencies were expressed as percentages. Independent t-tests were used for the comparison of continuous variables measurements, while the chi-square test was for categorical variables. Multivariable logistic regression analysis was done to study the association between cannabis users and HCC. The regression model was adjusted for demographics (age, race, and gender) including Elixhauser co-morbidities, and other relevant co-morbidities (HBV, HCV, and chronic liver disease). A P-value of less than 0.05 was considered statistically significant. SPSS version 25.0 software (IBM Corp, Armonk, NY) was used for all statistical analyses.

## Results

Study population

Of the 101,231,036 adult patients in NIS from 2002 to 2014, we identified 996,290 (1%) patients with cannabis use, compared to 100,234,746 (99%) patients with no cannabis use. Table 1 describes patients’ characteristics stratified by the presence of cannabis use. Cannabis-user patients were younger (mean; 34 vs 48), more males (61.7% vs 41.4%) and had more African American patients (29.9% vs 14.2%) compared with the control group, (P < 0.001 for all). Cannabis users group had a higher prevalence of alcohol abuse, smoking history, HBV, HCV, and liver cirrhosis. Meanwhile, they had a lower prevalence of HCC, obesity, DM, and gallstones compared to non-cannabis user group (P < 0.001 for all).

**Table 1:** Baseline characteristics comparison of cannabis abuse and non-cannabis abuse patients Abbreviations: SD: standard deviation; HCC: hepatocellular carcinoma; HBV: hepatitis B virus; HCV: hepatitis C virus; NAFLD: non-alcoholic fatty liver disease; DM: diabetes mellitus

Variable	Cannabis abuse 996,290 (1%)	Non-Cannabis abuse 100,234,746 (99%)	P-Value
Age (mean ± SD)	34± 13.24	48± 27.99	<0.001
Female %	38	59	<0.001
Race %			<0.001
White	55.6	66.3	
Black	30	14	
Hispanic	9.6	12.8	
Asian or Pacific Islander	0.8	2.7	
Native American	1.1	0.6	
Other	3.1	3.4	
HCC %	0.07	0.1	<0.001
HBV %	0.5	0.2	<0.001
HCV %	5	1	<0.001
NAFLD %	0.4	0.4	<0.001
Liver cirrhosis %	1.4	1.2	<0.001
Smoking %	44	9	<0.001
Alcohol abuse %	28	3	<0.001
Obesity %	6	7	<0.001
DM %	6	14	
Gallstones %	0.3	0.5	<0.001

We also identified 111,040 (0.1%) patients with HCC, of which 734 (0.7%) patients were cannabis users. Table 2 describes the HCC baseline characteristics stratified by cannabis abuse. Patients with HCC who were using cannabis were younger (mean; 55 vs 60), more male (87.2% vs 72.1%), and had more African American patients (26.9% vs 14.2%) compared to HCC patients who were not using cannabis, (P < 0.001 for all). HCC patients who were cannabis users had a higher prevalence of alcohol abuse, smoking history, HCV, NAFLD, and liver cirrhosis and a lower prevalence of DM, (P < 0.001 for all). History of liver transplants was lower in patients with HCC and cannabis users compared to those with HCC and no cannabis use, however, the P-value was not significant. 

**Table 2:** Baseline characteristics of hepatocellular carcinoma patients stratified by cannabis abuse and non-cannabis abuse Abbreviations: SD: standard deviation; HBV: hepatitis B virus; HCV: hepatitis C virus; NAFLD: non-alcoholic fatty liver disease; DM: diabetes mellitus

Variable	Hepatocellular Carcinoma 111,040 (%)		P-Value
	Cannabis abuse 734 (0.7%)	Non-Cannabis abuse 110,306 (99.3%)	
Age (mean ± SD)	54± 8.89	60± 16.87	<0.001
Female %	12.8	27.9	<0.001
Race %			<0.001
White	52.8	55.5	
Black	26.9	14	
Hispanic	15.5	16.7	
Asian or Pacific Islander	0.0	9.4	
Native American	1.7	0.7	
Other	3.2	3.8	
HBV %	6.9	7.8	P=0.44
HCV %	73.6	36.3	<0.001
NAFLD %	0.4	1.5	P=0.01
Liver cirrhosis %	67.8	46.5	<0.001
Smoking %	41.7	10.3	<0.001
Alcohol abuse %	53.1	15.7	<0.001
Obesity %	4.2	4.2	P=0.99
DM %	22.6	30	<0.001
History of liver transplant %	0.3	0.7	P=0.19
Iron overload %	0.5	0.3	P=0.15
A1 antitrypsin %	0	0.1	P=1.0

Cannabis users-HCC association

Using multivariable logistic regression, and after adjusting for patients’ demographics, co-morbidities, and hospital characteristics, cannabis users were 55% less likely to have HCC (OR, 0.45, 95% CI, 0.42-0.49, P < 0.001) compared to non-cannabis users.

Variables associations

Using multivariable logistic regression, HBV (OR, 3.62, 95% CI, 3.52-3.71, P < 0.001), HCV (OR, 5.9, 95% CI, 5.79-6.01, P = 0.001), chronic liver disease (OR, 14.53, 95% CI, 14.25-14.8, P < 0.001), alcohol abuse (OR, 1.44, 95% CI, 1.41-1.46, P < 0.001), iron overload (OR, 3.38, 95% CI, 2.98-3.83, P < 0.001), A1 antitrypsin (OR, 3.15, 95% CI, 2.5-3.95, P < 0.001) were more likely to have HCC. Meanwhile, females (OR, 0.39, 95% CI, 0.38-0.4, P < 0.001), smoking (OR, 0.62, 95% CI, 0.61-0.64, P < 0.001), and obesity (OR, 0.64, 95% CI, 0.62-0.66, P < 0.001) were less likely to be associated with HCC (Table 3).

**Table 3:** Multivariable regression analysis for the factors associated with hepatocellular carcinoma Abbreviations: OR: odds ratio; CI: confidence interval; HBV: hepatitis B virus; HCV: hepatitis C virus; NAFLD: non-alcoholic fatty liver disease; DM: diabetes mellitus

Variable	OR (95% CI)	P-Value
Race (compared to White)		<0.001
Black	1.24 (1.21-1.26)	
Hispanic	1.53 (1.51-1.56)	
Asian or Pacific Islander	4.97 (4.85-5.09)	
Native American	1.14 (1.06-1.24)	
Other	1.51 (1.46-1.56)	
Female	0.39 (0.38-0.4)	<0.001
Cannabis abuse	0.45 (0.42-0.49)	<0.001
Obesity	0.64 (0.62-0.66)	<0.001
Alcohol abuse	1.44 (1.41-1.46)	<0.001
DM	1.23 (1.21-1.24)	<0.001
HBV	3.62 (3.52-3.71)	<0.001
HCV	5.9 (5.79-6.01)	<0.001
Chronic liver disease	14.53 (14.25-14.8)	<0.001
Iron overload	4.7 (4.15-5.32)	<0.001
A1 antitrypsin	3.15 (2.5-3.95)	<0.001

## Discussion

Our study explored the 2002 - 2014 NIS dataset to evaluate the association between HCC and cannabis use among discharged patients aged 18 years and above in the USA. To our knowledge, this is the largest study in the literature evaluating the relation between cannabis use and HCC. In our analysis of nearly 1 million cannabis users, we found that patients with a history of cannabis use have a lower likelihood of having HCC. The demographic data of our cannabis users dataset was similar to previously published literature [11]. Cannabis users tended to be younger with a mean age of 34 years compared to non-users with a mean age of 48. They were more likely to be men, with 62% of users identifying as males. There was also a disproportionate representation of African Americans, with over 30% of cannabis users identifying as black.

Not surprisingly, cannabis users had a higher tendency to engage in higher-risk behaviors, including alcohol abuse 28% vs only 3% among non-users, smoking 44% vs 9% among non-users. Moreover, chronic viral hepatitis was more prevalent among cannabis users with HBV infections prevailing around 0.5% compared to 0.2% among non-users and HCV infections prevailing around 5% vs 1% among non-users. Although we do not have clear evidence, we presume the higher rates of viral hepatitis can possibly be attributed to a high-risk behavior such as intravenous illicit drug use where syringe sharing is common. Nonetheless, the prevalence of liver cirrhosis was relatively similar in both groups averaging around 1.2-1.4%. It is worth noting that we found that the prevalence of NAFLD did not differ among cannabis users averaging 0.4% in both groups. This is a rather surprising observation as it directly contradicts findings reported by Adejumo et al. who concluded that the prevalence of NAFLD was 15% lower in occasional cannabis users and 52% lower in heavy cannabis users [12]. Although their patient population was also extracted from the NIS database, we reason that their study could have possibly been limited by its population size as they only addressed hospitalizations in 2014.

Our study also confirms previously established risk factors for HCC. We found that patients with HCV infection were 5.9 times more likely to have HCC, while patients with HBV infections had 3.62 higher odds of having HCC. Cirrhotic patients were almost 10 times more likely to have HCC compared to the general population. Additionally, Iron overload and A1 antitrypsin deficiency were both associated with higher odds of having HCC at 4.7 times and 3.15 times, respectively. On the other end, cannabis users were slightly less likely to be obese at 6% compared to 7% of non-users, which echoes similar findings reported in previous studies [12,13]. Recent in-vivo studies also support that claim by showing that cannabis use reduces adiposity and prevents metabolic disease [14]. Additionally, we found that cannabis users were less likely to have type 2 diabetes mellitus, which backs similar reports by Loomba et al. [15] and Bellentani et al. [16]. In fact, there is evidence suggesting that cannabis use reduces insulin resistance [17]. 

Cannabis can exert its effect through two active chemical compounds, tetrahydrocannabinol (THC) and CBD which bind to their respective cannabinoid receptors CB-1 and CB-2 in the body [18]. The receptors belong to the family of G protein-coupled receptors and mediate the biological effects of phyto-derived and endogenous cannabinoids [19]. THC acts preferentially on CB-1, while CBD has a higher affinity for CB-2. Their activation results in the psychoactive effects of cannabis and are associated with pro-inflammatory effects. In contrast, CB-2 activation has no psychogenic effect but is rather anti-inflammatory.

In the liver, CB1 receptor signaling stimulates the hepatic stellate cells, thus promoting steatohepatitis and liver fibrosis, whereas CB-2 receptor activation decreases hepatic immune cells infiltration, Kupffer cell activation, oxidative stress, hepatic injury, and fibrogenesis resulting in inhibition of liver cirrhosis [20,21]. Naturally, the expression of cannabinoid receptors in the normal liver is low or absent [22]. On the contrary, up-regulation of cannabinoid receptors has been demonstrated in NAFLD, alcoholic liver disease, autoimmune and viral hepatitis, and cirrhosis [23]. Tetrahydrocannabivarin (THCV) and CBD directly reduced accumulated lipid levels in vitro in a hepatosteatosis model and adipocytes [24]. In another study, MDA19, a novel CB2 agonist, was found to inhibit the growth of hepatocellular carcinoma cells [25]. These studies collectively show that CB1 and CB2 have the potential to serve as therapeutic targets. In short, the CB-2 agonist activity exerted by cannabis, specifically CBD, offers an explanation to our observations by providing protection against HCC or at least deceleration of disease progression. Furthermore, pharmaceutical development of compounds exerting the dual effect of CB1 antagonism and CB2 agonism can play a major role in the management of liver diseases [26].

Limitations

The NIS Is an administrative database that primarily created for financial and administrative management rather than research purposes. Accordingly, the data collected may vary in the degree of detail and accuracy. Additionally, the NIS dataset as with other claims-based databases is limited by its cross-sectional study design [27]. As a result, there is recall bias in reporting exposures, not well-established sensitivity and specificity of ICD-9-CM codes for cannabis use. The International Classification of Diseases-9 coding system is unable to determine with certainty if a particular diagnosis was made during the hospitalization of record or if a patient carries a history of such a diagnosis. Thus, in patients with a history of cannabis use we cannot determine whether they are actively using cannabis or merely have a remote history of use. Also, co-morbidities were extracted based on ICD-9-CM codes; and therefore, severity and duration of co-morbidities could not be accounted for. There is also concern for under-reporting of cannabis use due to attached stigma. Finally, the study does not study the direct use of CBD but rather cannabis due to the absence of a specific ICD-9 code. 

## Conclusions

To the best of our knowledge, this is the first and largest population-based cross-sectional study of hospitalized patients to explore the association between cannabis use and HCC. Our analysis revealed that cannabis users were 55% less likely to have HCC compared to non-cannabis users. Due to the cross-sectional structure of our study, we are unable to draw direct causation effects. Hence, we suggest prospective clinical studies to further understand the mechanism by which various active ingredients, particularly CBD in cannabis, may possibly regulate hepatocellular carcinoma development.

## Appendices

**Table 4:** List of Elixhauser comorbidities included in the analysis

Elixhauser Comorbidity
Diabetes mellitus
Alcohol abuse
Obesity
Chronic liver disease

**Table 5:** ICD-9-CM codes for identifying HCC, cannabis, and other risk factors

Clinical condition	ICD-9-CM codes
HCC	155.0
Cannabis abuse	304.3, 304.30, 304.31, 304.32, 304.33, 305.2, 305.20, 305.21, 305.22, 305.23
NAFLD	571.8
Hepatitis B	070.33
Hepatitis C	070.54
Liver cirrhosis	571.5
Gallstones	574
Tobacco abuse	305.1
Iron overload	275.03
A1 antitrypsin	273.4
